# Feasibility of Digitally Identifying and Minimizing Stressors in Palliative Care Workplaces by Measuring Stress Continuously for Nurses Through Wearable Sensors (DiPa): Protocol for a Prospective Cross-Sectional Study

**DOI:** 10.2196/63549

**Published:** 2025-07-16

**Authors:** Aaron Seehausen, Wencke Chodan, Florian Höpfner, Carolin Schneider, Sabine Felser, Hugo Murua Escobar, Mario Aehnelt, Christian Junghanss

**Affiliations:** 1 Clinic III – Hematology, Oncology, Palliative Medicine Rostock University Medical Centre Rostock Germany; 2 Fraunhofer Institute for Computer Graphics Research Rostock Germany

**Keywords:** stress, wearables, psychological distress, psychological health, work-life balance, smartphones, ecological momentary assessment, workplace mental health

## Abstract

**Background:**

Nursing in palliative medicine combines primary patient care with the special challenges of this medical field (eg, handling the processes of dying, grief, and death). These cause high stress levels and burden on the nursing staff, resulting in an early exit from working life because of physical or psychological disorders like burnout.

**Objective:**

DiPa (digitally identifying and minimizing stressors in palliative care) is a prospective study investigating the feasibility of measuring burden and its causes in palliative care using methods of subjective and objective stress detection. Based on these results, stress-reducing interventions are to be deduced and evaluated. In this paper, we present our study protocol.

**Methods:**

The nursing staff of an inpatient university palliative hospital ward gathered data over 6 weeks. Each was equipped with a smart wristband and a smartphone that continuously measure physiological and ambient parameters throughout their working day. These objective data were enriched by subjective measurements: a questionnaire at the beginning of the study that assessed multiple potential stressful situations and constellations in the private and working environment as well as ecological momentary assessments (EMAs) during the workday. The EMAs were prompted by scanning near-field communication (NFC) tags placed at different locations on the ward. The ongoing data analyses will be processed using computer algorithms partly programmed specifically for this study and partly drawn from existing libraries, such as toolboxes for neurophysiological signal processing for Python. Comparisons between subjective and objective measures and group comparisons between variables of interest will be made using inferential statistics, including regression analyses and analyses of variance. Data analysis using machine learning algorithms will be implemented once sufficient data are gathered.

**Results:**

The study was funded in October 2019. As of July 2025, 12 of 18 nurses in the palliative care unit consented to participate in our study. We expect to start detailed data analysis in in the third quarter of 2025 and to finish and publish our results in 2026.

**Conclusions:**

The DiPa study aims at testing the feasibility of measuring and merging subjective and objective stress parameters for palliative care nurses.

**Trial Registration:**

German Register for Clinical Studies DRKS00024425; https://drks.de/search/en/trial/DRKS00024425/details

**International Registered Report Identifier (IRRID):**

DERR1-10.2196/63549

## Introduction

### Background

Chronic and severe diseases are increasing in importance and require sufficient medical treatment and well-educated specialists in the field to provide medication, help with personal hygiene, and offer counselling. In 2020, 1.7 million nursing staff worked in nursing and older adult care in Germany [1], which reflects a growing shortage of skilled workers in view of the aging population. The reasons are manifold, but the reasons found in the working environment include the 3-shift system, time pressures, and heavy physical and emotional burden, leading to dissatisfaction or even leaving the profession [2,3]. These factors can be found in almost all hospital departments but are exacerbated in areas with a high level of physical and interpersonal care. Palliative medicine is one of these particularly sensitive areas.

### Palliative Medicine

Palliative medicine is a specialty for the treatment of people with critical diseases like advanced cancer or higher-grade cardiac, renal, or lung diseases with limited life expectancy. Palliative care is a challenging discipline, not only due to the required medical care but also due to the psychosocial and spiritual aspects in the interaction between nursing staff and patients or their relatives, especially given the aim of improving quality of life in the last months and weeks of the patients’ lives. The main tasks of nursing staff in the palliative field include life support for the terminally ill and relieving their suffering from commonly experienced severe symptoms. These include pain, shortness of breath, and nausea, as well as anxiety and depression, which are also highly prevalent, as Sewtz et al [4] showed in their study.

### Burden in Palliative Caregiving

Palliative nurses face many work-related stressors (like interruptions, time pressures, interpersonal conflicts, or shift work, as aforementioned) that are also experienced by other professions in addition to stressors that are unique to taking care of patients who are terminally ill or close to dying. Additionally, they are not able to express their needs, which can also lead to burden as Seibel et al [5] have published in 2024 (see Table 1, adapted from [6]). It is an imminent part of the job—physicians and palliative nurses are confronted with the end of a patient’s life and with patients and relatives facing this stage. Even though nurses in this field choose this sensitive job after careful consideration, it still is an emotional burden to cope with the dying and death of patients for 54% to 68% of nurses in palliative care [6], which is moderated by circumstances (eg, age of the patient, patient and caregiver agony).

**Table 1:** Examples of potential stressors in a palliative care unit (PCU).

Factors	Examples
Team-associated factors	Staff fluctuation among physicians in the PCU Lack of support Lack of appreciation by other professional groups
Patient-associated factors	Young patients’ ages High care effort due to symptoms (eg, wounds, nausea, breathlessness, restlessness) Insufficient symptom control (eg, therapy-resistant pain, insatiable bleeding)
Structural factors	Understaffing High amount of documentation Inadequate education of patients and relatives about the palliative setting by the referring doctor
Personal factors	Ethical conflicts Feeling of being overwhelmed High expectation of one’s own work

Only a few studies have investigated this topic [6-12]. Diehl et al [11] surveyed and compared strains and resources for 149 nurses (34.5% response rate) working in the field of palliative care (in hospitals, hospices, or outpatient care in Germany). In their study, more than one-half of the nursing activities were perceived as heavy burdens, including transferring patients, time pressures, having to care for too many patients per day, handling patients with severe symptoms, and dealing with grief and death. Being surrounded by these topics every working day, nurses are at risk of psychiatric disorders that can accumulate to result in burnout syndrome in 15% to 20% of palliative nurses in home care [12,13].

Clinicians and researchers demand more studies, as urged by Pereira et al [10] in their systematic review of burnout syndrome in palliative care. Further evaluations of palliative care nurses throughout Europe could ensure better positioning of any results in the transnational context of the palliative care area and reveal further deficits in the care structure [9].

### Digitally Identifying and Minimizing Stressors in Palliative Care Study

The DiPa (Digitally Identifying and Minimizing Stressors in Palliative Care) study was conceptualized to use digital technologies (“Di”) to assess the working conditions of and burden on nurses in palliative settings (“Pa”). The study is a joint project of the Fraunhofer Institute for Computer Graphics Research (IGD) Rostock and the University Medical Centre Rostock (UMR) and funded by the State Office for Health and Social Affairs Mecklenburg-Western Pomerania. The study aims to identify correlations between measured objective physiological parameters and subjective perceptions of stress in everyday situations in the palliative care unit (PCU). Therefore, the primary goal is demonstrating the feasibility of identifying stressors in palliative care and measuring stress reactions with wearable devices. With these initial results, stressors and stress can be reduced by developing preventive measures.

### Physiological Stress Model

To explain our rationale for using wearable devices to measure stress in humans, we present the physiological stress model. The autonomic nervous system is responsible for all vital functions, regulating breathing, digestion, and the cardiovascular system as well as influencing the heart and glands [14]. The autonomic nervous system is divided into the sympathetic nervous system (SNS) and parasympathetic nervous system, which often act antagonistically. Although the SNS is activated by stress, the parasympathetic nervous system supports resting states. “Stress” occurs when the demands of the environment exceed the individual’s ability to cope [15]. It is defined as an individual’s reaction to burden and is caused by triggers that are called “stressors.”

Three phases of the physiological reaction can be distinguished. The phases were first described as part of the “general adaptation syndrome” by Selye and Köbcke [16]: The first phase is called the “alarm phase” and appears immediately after the stimulus.

The SNS reacts to a stressor by releasing catecholamines like noradrenalin and adrenalin from the adrenal medulla [17] and, simultaneously, adrenocorticotropic hormone from the cortical part of the brain [18]. In their paper, Chodan et al [19] described how the different physical changes interact during the alert: The muscles require oxygen for the oxidation of glucose and fat into energy. To provide this surplus of oxygen, breathing rate and heart rate (HR) increase. Empirical knowledge and experiences with handling data from biomedical parameters gathered with wearable devices have been accumulated in prior studies [19-21]. In particular, HR and heart rate variability (HRV) have been identified as valid stress markers [22,23].

To facilitate chemical reactions to provide energy (ie, the oxidization of glucose and fat), body temperature rises. At the same time, to protect the body from overheating, the sweat glands are also targeted by the SNS, increasing skin conductance and lowering skin resistance, which can be measured between 2 electrodes placed onto the skin [24]. Thus, measuring sweat secretion is an important method to investigate the activity of the SNS and to determine stress reactions.

Directly after the alarm phase, the “resistance phase” ensures and is biochemically characterized by increased cortisol liberation. Cortisol passes through the blood-brain barrier to the hippocampus, which then lowers the activity of the hypothalamic-pituitary-adrenal axis via negative feedback mechanisms. This serves to regulate stress reactions and prevent exhaustion of stress hormones.

Only in chronic stress (eg, when the available resources are insufficient to relieve stress or when the stressor is present over a long period of time), a third phase, “exhaustion phase”, follows, with symptoms of exhaustion and a dangerous weakening of the immunological system [25].

The DiPa study aims to test the feasibility of measuring and merging subjective and objective stress parameters in palliative care nurses.

In addition to physiological models of stress, various psychological frameworks have been developed to explain the conditions under which stress occurs in the workplace. One of the most widely known is the Job Demand-Control (JDC) model, originally proposed by Karasek [26]. This model posits that stress results from the interaction between the psychological demands of a job and the individual’s decision latitude, or control, over their work tasks. According to the model, high job demands combined with low control are particularly detrimental, leading to increased strain and adverse health outcomes.

It is important to acknowledge that the JDC model—and other cognitive-behavioral stress frameworks—can be meaningfully integrated with our somatic approach. All major stress models, despite differing in their conceptual emphasis (eg, cognitive appraisal, social support, or organizational structure), converge on the notion that stress elicits a biological stress response. Our focus on this common somatic end point allows for alignment with a broad range of theoretical models, including the JDC model. Specifically, job conditions characterized by high demands and low control, as described in the JDC framework, are likely to be present in the workplace of palliative care nurses and are thus likely to activate the very physiological stress pathways that are measured in this study.

## Methods

### Study Design

To assess the working conditions of and burden on nurses in palliative settings, preliminary tests were executed to investigate the functionality of and user experience with wearables, including handling in a hospital environment and resistance to the hospital’s disinfection routines.

**Figure 1:**
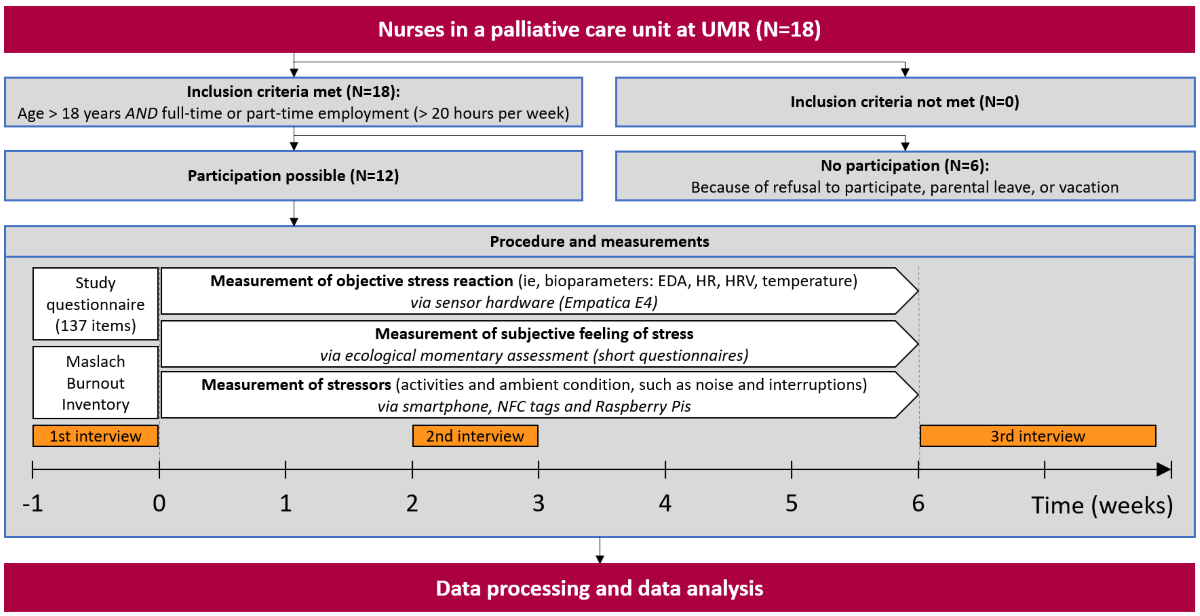
Study design. EDA: electrodermal activity; HR: heart rate; HRV: heart rate variability; NFC: near-field communication; UMR: University Medical Centre Rostock.

The study design (Figure 1), a prospective cross-sectional approach with continuous measurements throughout each workday, allows for assessing both the current manifestation of stressors and current level of perceived stress load both objectively and subjectively. Participants were split into groups, and each group conducted the measurements for 6 weeks, thus expanding the span of the measurement across all participants (to increase the time frame depicted in the data), while individual participants were not burdened with the additional workload for longer than necessary.

### Apparatus

#### Overview

Stress level was measured both objectively (physiological stress reaction) and subjectively (appraisal of the perceived stress in ecological momentary assessments [EMAs]). Possible stressors were also identified both as subjective perception via questionnaires and objectively by matching physiological stress responses to concurrent ambient conditions, such as noise, interruptions, or specific activities. We compared the objective stress levels to the daily staffing, occupancy level, and other daily changing factors of a hospital ward.

**Figure 2:**
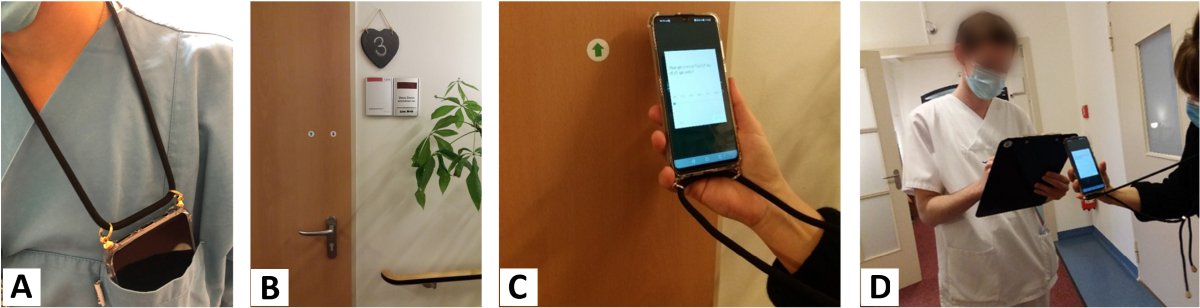
To assess possible stressors, near-field communication (NFC) tags have been programmed and placed in numerous positions on the ward.

**Figure 3:**
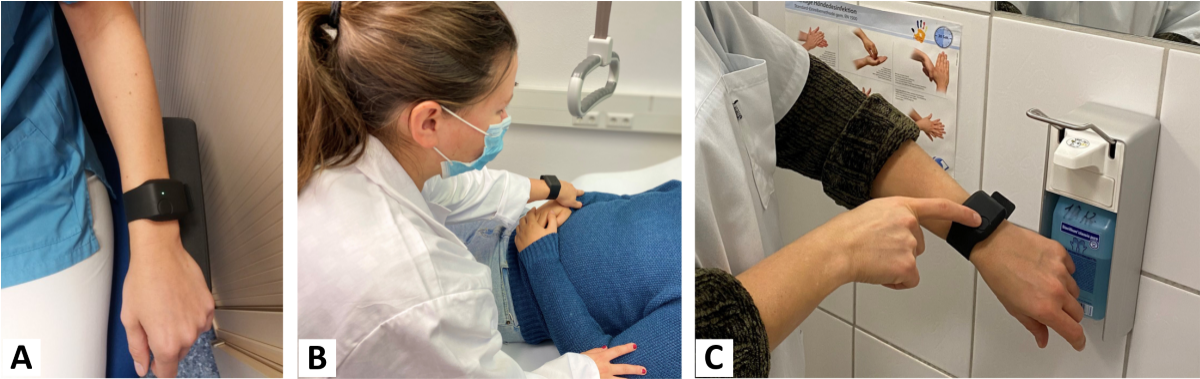
Electrodermal activity, heart rate, and skin temperature are recorded with the Empatica E4 research wristband.

To this end, a combination of wearable and ambient sensors that can measure the physiological (objective) stress reaction as well as possible stressors (causes of stress) is being used. The prototypical setup consisted of a smart wristband, a smartphone, near-field communication (NFC) tags, and Raspberry Pis equipped with microphones to record interruptions (incoming telephone calls) and unexpected tasks (calls from patients). The setup can be seen in Figure 2 and Figure 3. At the end of the study, it will be possible to characterize a normal working day of a palliative caregiver more precisely in terms of physical and psychological stress. The perceived burden will be correlated with bed occupancy and the numbers of patient admissions, discharges, and deaths per day as well as influence of the duty roster design (including the staffing) and frequency of interruptions by telephone or a patient’s bell.

#### Body-Worn Equipment

The results of measured electrodermal activity (EDA), HR, and skin temperature should provide information about an SNS reaction to stress [14]. All these parameters are recordable with the Empatica E4 (Empatica Inc) research wristband. Thus, to measure the physiological stress reaction, we used the commercially available Empatica E4 wristband [27]. The E4 wristband possesses a photoplethysmography sensor and 2 electrodes to measure the galvanic skin response at the wrist. It was used to measure pulse, HRV, EDA, and body temperature (surface). The automatic data preprocessing was bypassed, since this would require sending the sensitive data to an alien server. To circumvent these international servers for reasons of data protection, the code of the E4 had been altered, allowing the extraction of raw data. Raw data were then sent to the smartphone, which is also part of the study’s setup, and stored on the phone’s memory until transferred to a local server. The E4 had no display and thus provided no feedback to the participants that could interfere with or distort the measurement. LEDs provided the necessary feedback on the functionality of the wristband, and additional feedback on functionality (and connectivity) was given in the corresponding smartphone app.

In addition to the LED, allowing event marking (ie, a real-time annotation) via tapping the button deposits a record in the session file. The record included the time of the button press (expressed as a Unix time stamp in UTC), which was synchronized with the initial time of the corresponding session. Feedback on the button press was given both visually (illuminating the LED) and audibly (beeping). The button press was used for 2 purposes: (1) logging an event of subjectively perceived stress (a single button press) and (2) logging the event of disinfecting one’s hands (as this can alter the EDA; 2 consecutive button presses).

#### Preparation of the Hospital Ward

To assess possible stressors, NFC tags (Figure 2C) had been programmed and placed in numerous positions on the ward. The NFC tags were used to log the activity of the participants, such as NFC tags placed on the doors of the 9 patient rooms to log when a participant entered or left a specific patient room. Other activities that were logged included the beginning and ending of meetings (medical rounds; daily, weekly, and monthly team meetings; and daily handovers at the beginning or end of a shift), the beginning and ending of conversations with patients or their relatives, and relaxation.

Furthermore, in addition to pressing the wristband button, as aforementioned, a stressful event could also be marked by scanning the NFC tags, which offered the possibility to communicate a challenging situation by entering a short description of the current event using a Huawei smartphone P30 lite. This smartphone, which hangs by a cord around the participants’ necks and can be stored conveniently in their gowns’ pockets with the microphone poking out (see Figure 2A), was also used to measure ambient noise.

Last, 2 types of interruptions were measured, each with a microphone attached to a Raspberry Pi 4 Model B. The first was an interruption by telephone calls (eg, outsiders calling to reach patients or to retrieve information about the patients such as family physicians or calls from within the hospital, eg, calling about referrals). The second kind of interruption was a distress call (from a patient) or an emergency call (from a fellow nurse or physician), both broadcast through the hospital ward’s bell system.

#### Questionnaires

Two types of questionnaires were used to assess subjective stress levels. An extensive study questionnaire with 137 items (101 of which ask for specific stressors) was handed out at the beginning of the study. It asked about the subjectively perceived frequency and participant’s rating of different stressors (ie, how often does this event take place and how stressful does one find it). A multiplication of the 2 parameters allowed for an evaluation of the perceived stress. Although an event could be labeled as very stressful, it was not necessarily a meaningful stressor if it rarely ever occurred. Thus, a stressful event that seldom took place or a frequent event that barely stressed the participant were both considered less harmful than an event that was both frequent and (moderately or highly) stressful. The study questionnaire was designed specifically for this study by a psychologist and a nurse with longstanding work experience, incorporating known stressors from the literature as well as stressors mentioned in a survey carried out in a palliative ward and an outpatient palliative care facility.

In addition, the Maslach Burnout Inventory [28] was handed out to assess further burdens (or possible sequelae of the stress). It is a standard questionnaire that will allow for interstudy comparisons of this study’s findings with those in the literature.

In addition to these 2 questionnaires that were only completed once at the beginning of the measurements, the subjective stress level was also assessed throughout the workday and accompanied the continuous ambulatory measurements using EMAs. EMAs are short questionnaires asking for self-reported information that documents the perceived subjective experience of stress at a certain moment (ie, a *momentary* appraisal of their *current* stress level) [29]. The questionnaires popped up on the participants’ smartphones whenever an NFC tag was scanned that indicated the end of an activity (eg, end of rounds, leaving a patient room, end of a meeting, end of speaking with a patient or his or her relatives) and asked, for example, “How stressed do you feel right now?” with a continuous slider from 0% to 100%, labeled in increments of 20%. In addition, the questionnaire could also be accessed at any time by scanning a designated NFC tag called “open questionnaire.” Additional EMAs popped up when the participant put the setup on at the beginning of a shift (greeting the participant, asking for the subjective sleep quality of the night before, and asking about his or her current stress level) and when the setup was removed and shut down at the end of the shift (asking for the current stress level, asking how many patients were looked after throughout the day, and wishing the participant a pleasant recreational time).

### Sample and Recruitment

The PCU at UMR, with its 14 beds, is the largest PCU in Mecklenburg-Western Pomerania, Germany. It employed 13 full-time nurses and 5 part-time nurses who work in a 3-shift system during the study implementation in 2021. Study participants had to be older than 18 years, their working time had to have a minimum of 20 hours per week, they had to have proficiency in written and spoken German, and they had to be employed at the palliative care unit at the Rostock University Medical Centre (inclusion criteria). Exclusion criteria comprised of nonconsenting capacity and nonfulfilment of all inclusion criteria. For the DiPa study, all 18 nurses working in the UMR PCU, who partly specialize in palliative care, were invited in December 2020 to participate in the study by presenting the procedure and aims of the project in a lecture as well as in a written inquiry. The measurement period for each participant was 6 weeks allowing for a total measurement duration of 3 months.

A power analysis was not conducted as the population was prespecified (N=18), rendering the need for an accruing sample. In the end, 12 nurses agreed to participate, resulting in a participation rate of 67% (12/18).

### Patient and Public Involvement

Patient involvement was not directly part of our study design because of the focus on nursing staff in palliative care. However, analogous to patients, our study participants were included in selected questions when we created the study design. As an example, preliminary tests could be used to compare measuring instruments with different measuring methods with regard to their wearing comfort and manageability. Furthermore, before starting the measurements, the nursing staff were shown a list of possible stressors in their work area. Anyone who had an additional suggestion could add it anonymously to this list. After the study design was developed further, the study was presented to the potential participants in an on-site presentation, as aforementioned. There was also the opportunity for questions and answers. Each nurse was free to take part in the study after having detailed knowledge of the study objective and carrying out the measurements on a daily basis.

Apart from our study participants, the public was not especially included in the planning phase. The expected results could affect the public and especially nurses in palliative care or other medical departments indirectly by identifying stress factors and their possible reduction.

### Procedures

After enrollment, each participant received thorough instruction on how to use the technical equipment. On the same day, each participant was interviewed separately to define their expectations and attitudes toward the DiPa study. Interviews were conducted by UMR staff who were not involved in the study (ie, a research assistant or a member of the central research center of the UMR). The answers were recorded during the interview and transcribed afterward to allow for an evaluation by independent raters to reduce bias and ensure data privacy. In addition, the participants completed the questionnaire on their perception of stressors and stress, including patient-associated stressors, working environment, team-associated stressors, and resources.

Participants were instructed to conduct the measurements during every shift. At the beginning of the shift, the participants gathered their equipment from their locker, put on the wristband, and picked up the smartphone. By turning on the wristband, a digital questionnaire appeared automatically on the smartphone’s display, welcoming the participant and asking for their current stress level as well as sleep quality of the previous night. Further free-text annotations were also possible. Participants then stored the smartphone in the pocket of their scrubs and began their workday.

Participants were instructed to use the smartphone to scan the corresponding NFC tag whenever entering a patient’s room. To this end, a green NFC tag with an arrow was placed on each patient room door. This event was thus recorded on the smartphone. Analogously, a red NFC tag was scanned when leaving the room. This not only recorded the event but also prompted a short questionnaire that was displayed on the smartphone’s screen, asked for their current stress level, and included a free-text space.

Special recurring processes on the PCU like daily visits, weekly and daily team meetings attended by nurses and physicians, and patient interviews had their own individual NFC tags denoting the start and end.

In parallel to data recording by the wristband and smartphone, the study team collected and analyzed data about the nurses (eg, how many worked in the ward per shift, length of work experience in years, and if they had the additional designation of “palliative care nurse,” as we expected an influence of workload for each participant). The second focus was patient-associated factors, especially the average, minimum, and maximum patient age and number of occupied beds per day. Because the PCU treats many critical ill patients with life-limiting diseases, we also thought it could be important to analyze the number of deaths per day.

### Data Analyses

Every participant received a pseudonym so that data from the different sources (interviews, questionnaires, physiological data from the E4, EMAs from the smartphone) can be grouped. Raw data analyses and analyses of data on an individual level are being conducted by the Fraunhofer IGD, an institution that is independent of UMR, ensuring data privacy. The UMR will receive the preprocessed, averaged data for further evaluation processes.

The experimental setup consisted of a local server that, via a locally installed network, only ensured the station’s data flow by transmitting data from smartphones and the wristbands. Local access to the server was limited by password-protected log-in. Data were also stored in pseudonymized form, so that no clear names could be found in the files. Data were automatically forwarded from the smartphone once a day to a server within the UMR via Wi-Fi connection. There, the collected information initially remained locally on the hard drive. At regular intervals, a Fraunhofer Institute employee brought along an encrypted data storage device on which he or she created a local backup copy on and transferred it to a computer within the Fraunhofer Institute. This prevented unauthorized access by unauthorized people.

The participants were informed in advance about the guarantee of data security, including the scope of the data collected and the duration of data storage, in their written consent form. These aspects were explained to an ethics committee and confirmed by a positive ethics vote.

The raw data were collected with the Empatica E4 device and stored in CSV files. Each parameter was sampled at the maximum available rate provided by the Empatica E4 firmware. The parameters included interbeat intervals (IBI) sampled at 1/64 second, blood volume pressure sampled at 64 Hz, EDA sampled at 4 Hz, temperature sampled at 4 Hz, and acceleration sampled at 32 Hz.

All parameters were stored with a Unix time stamp to allow the proper selection of intervals.

The EDA data were decoupled using the NeuroKit2 Python library so that the tonic component (skin conductance level) and the phasic component (skin conductance response) could be extracted.

Since the Empatica E4 wristband saved IBI only when the signal quality is sufficient, IBI data can include missing values. The IBI data were used to determine the HRV and HR by applying the following equation:



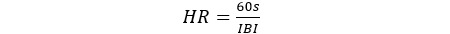



The acceleration data were fused to a 3D vector by applying the following equation:



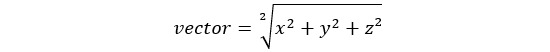



All algorithms for data extraction were written in Python using Jupyter Notebooks and the *Pandas*, *Numpy*, *SciPy*, *Matplotlib*, and *NeuroKit2* libraries.

For stress detection analysis, all stress events marked with a button press will be identified, and windows surrounding this event will be extracted and averaged, leading to one value per stress event per participant. Analogously, the windows surrounding a scan of the NFC tag “relaxation” and the baseline windows will be extracted and averaged as well. Descriptive and inferential statistical analyses will be applied using IBM SPSS Statistics 30. Weighted ANOVAs for correlated samples will be computed to compare the stressful events and the baseline for each physiological parameter.

To examine the predictability of experiencing stress, the subjective ratings given in the EMA will be used as ground truth. Surrounding the EMA, data windows will be extracted from the data for each physiological parameter. A linear regression will be computed with the subjective stress level (EMA) as the dependent variable and entering the means of the physiological variables (HR, HRV, tonic EDA, phasic EDA, temperature, and acceleration) as independent variables. Artificial intelligence approaches will be used to analyze the data where appropriate.

With the data for daily occupancy of patients and the total number of nurses on the same day, we will be able to calculate the relationships of these 2 variables with stress. The minimum occupation level according to the recommendation from the German Society for Palliative Medicine (*Deutsche Gesellschaft für Palliativmedizin*) on staffing in palliative wards [30] of 1.4 nurses per patient will be used as a cutoff to create two subgroups of “well-staffed” and “under-staffed.”

### Usage of Study Guidelines

We structured our study protocol using the SPIRIT (Standard Protocol Items: Recommendations for Interventional Trials) guidelines [30].

### Ethical Considerations

The ethics committee of the University of Rostock approved the study (approval A 2020-0295). Data protection is ensured by providing participants with their own lockers to store their devices, gathering data using pseudonyms only, and analyzing raw data at the Fraunhofer IGD exclusively (sharing only group means with the UMR, which employs the participants).

All possible participants received information in writing assuring them that participation was voluntary, that the choice of not participating would yield no harmful consequences, and that they could withdraw from the study at any time if they no longer wished to participate. Each participant had the possibility to ask further questions. They were asked by a study nurse, so the inviter and the participant were on the same hierarchy level, lowering any possible pressure that the nurses might have felt to participate. They were assured in speech and writing that they did not have to fear professional consequences or disadvantages if deciding against participating in the study.

## Results

The DiPa study was funded in October 2019; received ethics approval on December 18, 2020; and was registered in the German Register for Clinical Studies on February 10, 2021. As of January 2021, the devices were assembled, and the stationary equipment (eg, the server, the NFC tags) was placed on the ward. Recruitment to the trial is finished, with a total of 12 participants having signed the written consent form by June 12, 2021. The measurements were initiated by giving out the first questionnaires.

The data assessment algorithms have been programmed, and the data assessment has been finished.

We expect to finish data analysis and publish the final results at the end of 2026 or beginning of 2027. We aim to publish the data and summary findings in academic journals and at conferences. For the participants, these will be presented accessibly in a staff meeting.

## Discussion

### Potential Outcomes and Impact

We aim to achieve results that provide deeper insights into the stress levels of nursing staff on the palliative care ward. A central focus is on identifying the causes of this stress. An essential part of the study deals with the investigation of various stressors, which include patient care, contact with relatives, working conditions, and the collegial working environment. Based on the studies by Diehl et al [6,11,31-33], we assume that the challenging task of dealing with seriously ill, dying patients and their death is a significant source of stress. In addition, based on the preliminary discussions with the participants, we expect that a considerable part of the stress is triggered by contact with the patients’ relatives. As a third important factor, we assume that the working conditions, including duty rosters, interaction with colleagues, and the number and timing of meetings, have a significant influence on the participants’ experience of stress. In order to record these and other relevant factors such as air quality or activities, a complex test design is required. Although questionnaires can ask about subjective feelings at a particular point in time, they are not able to take sufficient account of the day’s events as a whole or capture aspects that the participants may not be aware of—a well-known problem with the use of questionnaires.

To our knowledge, the DiPa project is the first innovative project addressing the burden on nursing staff in palliative care using wearable devices to collect objective data in combination with subjective measurement instruments such as questionnaires.

Since this project is the first of its kind, it aims to measure the feasibility of generating data with the abovementioned technology as a primary outcome. As secondary outcomes, we want to identify correlations between the recorded bioparameters and parameters of the work environment (time, telephone, temperature, etc) as well as subjective well-being, especially in handling sensor technology in a normal working day—which could be a potential stressor by itself.

### Strengths and Limitations

The content-related strengths lie mainly in two aspects: the multitude of measurements and the continuous measurement. As for the multitude of measurements, the study can assess all three aspects of the stress phenomenon: the stressors that elicit stress (activities, ambient conditions, noise, and interruptions—measured via smartphone, NFC tags, and Raspberry Pis), the stress reaction in the sense of subjective appraisal and feelings (measured via EMAs on the smartphone), and the stress reaction in the sense of the objective stress reaction (bioparameters, EDA, heart rate, HRV, and temperature measured via sensor hardware).

In addition, the study also features a methodological strength in the unobtrusiveness, interaction, and automation of the measurement devices: the smartphone and the sensor hardware were worn by the participants and were easily accessed. The sensor hardware was linked to the smartphone and sent data automatically; the smartphone in turn sent these data automatically to the server during the times when it was stored and charged in the lockers. This allowed for smooth data acquisition and increased motivation of the participants to collect data over such a long period and while working.

The study apparatus, for example, the use of smartphones or the scanning of the NFC tags, introduces a potential for user fatigue or confusion. The feasibility of sustained use was not assessed in this study, as the study did and does not intend sustained use of the apparatus. The assessment was deliberately limited to a duration of weeks to gain insights on the status quo of perceived stress, with a long-term goal of deducing provisions to reduce stress. However, the level of perceived fatigue caused by the interaction with technological devices, as well as the level of acceptance of technology and skill level when interacting with technology, could be assessed in future studies with a larger sample to allow for analyzing potential influences of these factors.

In the same line, introducing repeated prompts during the workday (the EMAs)—together with the aforementioned use of technology—can act as a further stressor. While this method enabled us to capture real-time self-reports of perceived stress and contextual factors, it may have inadvertently increased participants’ cognitive load or interfered with task performance, thus acting as an additional stressor. This reactivity to the assessment process itself could potentially amplify or confound the stress responses we aimed to measure.

Nonetheless, EMA offers critical advantages that justify its use. Unlike retrospective questionnaires, EMA minimizes recall bias and allows for the assessment of stress as it unfolds in the natural work environment, providing high ecological validity. This approach also enabled us to capture dynamic fluctuations in stress levels across the workday and to relate them temporally to physiological stress markers. Importantly, the combination of EMA and physiological measures enhances our ability to understand the complex interplay between subjective experience and somatic stress responses.

Still, the potential reactive burden introduced by the method should be treated as a confounding variable. To mitigate its potential confounding effect, the EMA protocol was designed to be minimally intrusive. The number of mandatory EMA prompts were limited to two per shift: one at the start and one at the end of the workday. Additional EMA entries were linked to the scanning of the NFC tags, enabling context-specific data collection while reducing the need for separate prompts. These intermediate assessments were optional and could be skipped without consequence if participants felt rushed, overwhelmed, or otherwise unable to respond at the moment.

Furthermore, we actively monitored the acceptability and perceived burden of the EMA procedure through qualitative feedback. Participants were interviewed midway through the assessment period and again at the end, where they were asked about the extent to which the EMA prompts were experienced as disruptive or burdensome. While a detailed analysis of this qualitative feedback is beyond the scope of this paper, these data are being prepared for publication in a separate manuscript focusing specifically on participants’ subjective stress perception, including their experiences with EMA in high-demand clinical settings.

Regarding the study population, the final sample (n=12) includes no stratified analysis plan based on shift, age, or prior burnout, limiting subgroup insights as a result of the study design. There is also a self-selection bias risk. Participants volunteered, likely introducing selection bias; for example, more tech-savvy nurses participated. However, we do not believe that only more resilient nurses participated in the study. Indeed, it is likely that interest in the study results and the resulting reductions in stress led to increased participation among a group of nurses who demonstrated even lower levels of resilience to stress.

Nurse shortages and the high emotional and physical demands of palliative care contribute to chronic workplace stress, which can undermine both staff well-being and patient care quality. Yet, traditional methods of stress assessment (eg, surveys and end-of-shift reports) often fail to capture the real-time, fluctuating nature of stress in these settings. By using digital monitoring tools such as EMA and physiological sensors, we aim to better understand the moment-to-moment stress patterns nurses experience during their shifts. This approach not only captures stress more accurately but could inform future targeted interventions or adaptive support systems, especially in the strained setting of palliative care where staffing and resilience are critical. The effects of these targeted interventions could then be evaluated by using similar methods to ensure comparability while adding measures for practical relevance such as stress-related attrition (eg, burnout or leaving the profession).

### Future Directions

In the near future, the results are to be used to derive preventive measures to reduce stress. While it is a scientific end in itself to gain knowledge on the stressors, we would like to use this knowledge to help enhance the working conditions and the well-being of the nursing staff.

For example, structural processes can be questioned and adjusted to reduce stress and its impact. The effectivity of these interventions will be part of further studies.

Parameters that measure stress have trained a multidimensional model. Based on the known input parameters (HRV, tonic and phasic EDA, and NFC labels) and knowledge of their correlation with stress (from the literature), it is possible to determine which factors primarily influenced the decision or outcome by visualizing the activation of neurons within the network. For regression, the architecture of a fully connected (dense) neural network will be used, with the goal of building a regression model that targets a value between 0 and 100%.

## Abbreviations

DiPa: Digitally Identifying and Minimizing Stressors in Palliative Care
EDA: electrodermal activity
EMA: ecological momentary assessment
HR: heart rate
HRV: heart rate variability
IBI: interbeat intervals
IGD: Institute for Computer Graphics Research
JDC: Job Demand-Control
NFC: near-field communication
PCU: palliative care unit
SNS: sympathetic nervous system
UMR: University Medical Centre Rostock

## Data Availability

Because of our small number of study participants, it might be possible to draw conclusions about a particular person by publishing the detailed data. In our study consent form, the participants were informed about the data dissemination: “The data collected as part of the study will be processed and evaluated in cooperation with the Fraunhofer Institute. The data will only be passed on to other institutions or persons and published cumulatively (for example, averaged over the entire group of all participants), i.e. conclusions about individual persons are excluded.” Thus, we are not allowed to publish our data due to our own privacy policy.
